# Theoretical and Experimental Analysis of an Induction Planar Actuator with Different Secondaries—A Planar Driver Application for Metallic Surface Inspection

**DOI:** 10.3390/s16030407

**Published:** 2016-03-19

**Authors:** Felipe Treviso, Marilia A. Silveira, Aly F. Flores Filho, David G. Dorrell

**Affiliations:** 1 Post-Graduate Programme in Electrical Engineering, Federal University of Rio Grande do Sul, Porto Alegre, RS 90035-190, Brazil; aly.flores@ufrgs.br; 2 Electrical Engineering, Lutheran University of Brazil, Canoas, RS 92425-900, Brazil; marilia.amaral@ulbra.br; 3 School of Engineering, College of Agriculture, Engineering and Science, University of KwaZulu-Natal, Howard College Campus, Durban 4041, South Africa; dorrelld@ukzn.ac.za

**Keywords:** induction planar actuator, surface inspection, thrust force, normal force, analytical model, numerical model, measurement of force, linear induction motor

## Abstract

This paper presents a study on an induction planar actuator concept. The device uses the same principles as a linear induction motor in which the interaction between a travelling magnetic field and a conducting surface produces eddy currents that leads to the generation of a thrust force and can result in movement over a metallic surface. This can benefit the inspection of metallic surfaces based on the driving platform provided by the induction planar actuator. Equations of the magnetic and electric fields are presented and, by means of these equations, the forces involved were calculated. The behaviour of thrust and normal forces was analysed through the equations and by numerical models, and compared with the results obtained by measurements on a device prototype built in the laboratory as part of the study. With relation to the surface under inspection that forms the secondary, three cases were analysed: (1) a double-layered secondary formed by aluminium and ferromagnetic slabs; (2) a single aluminium layer and (3) a single ferromagnetic layer. Theoretical and measured values of thrust and normal forces showed good correlation.

## 1. Introduction

There are different types of electromagnetic planar actuators and they can be employed in various applications [1,2,3]. The inspection of metallic surfaces is one kind of application in which an induction planar actuator can be employed as a driver, even on leaning or curved surfaces. To do so, an adhesion principle can be employed. It can be based on principles such as magnetic, pneumatic, mechanical, electrostatic and chemical, and even on biologically-inspired robots [4]. This paper presents an induction planar actuator intended to be employed to drive a platform with a camera, tools and sensors suitable for the inspection of metallic surfaces, such as ship hulls, oil tanks and pipelines. In this kind of actuator, the surface under inspection assumes the role of the secondary. When there is a ferromagnetic material in its secondary, besides a thrust force, it produces an attraction normal force between the primary and the secondary. That normal force is responsible for the adhesion of the device on the ferromagnetic surface under inspection. On the other hand, that normal force cannot be so high that it prevents the movement of the actuator on that surface. While the generated normal force attracts the platform to the surface on which the actuator moves, the tangential component of the force produces the thrust that propels it.

Analytical and numerical analysis of forces that act on an electromagnetic actuator are important issues, as they allow an investigation of the device’s operation by quick means prior to the construction of prototypes. Many works present analytical models for analysis of the electromagnetic behaviour in DC [1] and AC [5,6,7,8,9,10] actuators. In this paper, the behaviour of the involved forces in the induction planar actuator is analysed by means of results obtained by analytical and numerical models and by measurements. With relation to the surface under inspection that forms the secondary, three cases were analysed: (1) a double-layered secondary formed by aluminium and ferromagnetic materials; (2) a single aluminium layer; and (3) a single ferromagnetic layer. Theoretical and measured values of thrust and normal forces showed good correlation.

## 2. The Induction Planar Actuator

**Figure 1:**
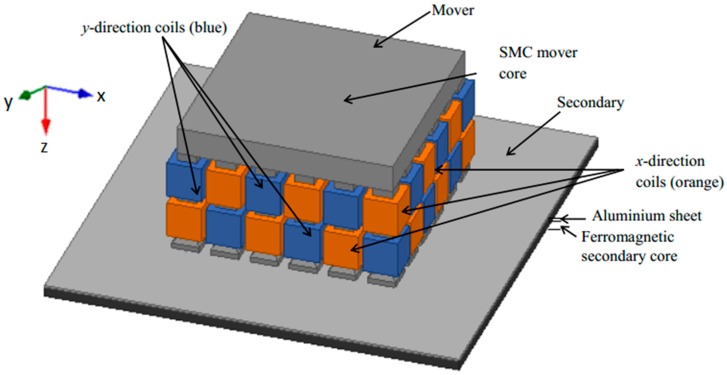
Double-layered planar actuator topology [11]. © 2015 IEEE. Reprinted, with permission, from IEEE Transactions on Magnetics.

The planar induction actuator under study is presented in Figure 1. It comprises two three-phase windings which are assembled in an orthogonal fashion with respect to each other. Each winding comprehends a set of coils that are distributed and placed around armature teeth made of SMC 1P700 Somaloy. The latter has a resistivity of 400 µΩm, and maximum relative permeability of 540 and is employed in the primary core because of its isotropic magnetic and electric properties. A lower magnetic loss in the material, when compared to solid steel, allows the reduction of ferromagnetic losses produced by the 3D magnetic field inside the core. There are two coils around each tooth of the core, and each such coil belongs to a three-phase winding responsible for generating thrust in the *x* or the *y* direction. The coils are placed in such a way that on one tooth an *x*-coil is mounted over a *y*-coil, and in the adjacent teeth, the *y*-coil is mounted over the *x*-coil, and so on, in order that both windings have the same number of coils in the upper and in the lower layer of coils. 

**Figure 2:**
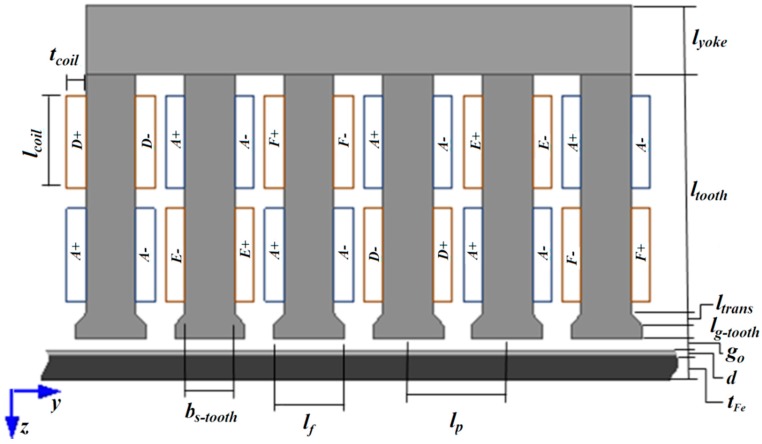
Construction dimensions of the induction planar actuator under study [11]. © 2015 IEEE. Reprinted, with permission, from IEEE Transactions on Magnetics.

The three cases of secondary surfaces analysed are composed of the same two materials: aluminium, with resistivity of 0.026 µΩm and maximum relative permeability close to the unit, and steel with resistivity of 0.1862 µΩm, and maximum relative permeability equals to 1400. In case 1, the authors employed a steel slab with a thickness of 5 mm with a 1-mm thick aluminium slab on top of it. In case 2, only the 1-mm thick aluminium slab is employed as secondary, and in case 3 the tests were carried out using only the 5-mm steel slab. Table 1 shows the description and values for the dimensions of the device, which are presented in Figure 2.

The windings produce travelling magnetic fields when the planar induction actuator is fed by three-phase currents. EMFs are induced into the secondary plate which produces electric current. The interaction between the travelling magnetic field and the eddy currents of the secondary produces two kinds of force: a thrust force in the direction of that travelling magnetic field and a normal force. The latter has a repulsive component due to the induced eddy currents in that secondary and it can have an attractive magnetic one when a high permeability steel plate is present in the secondary. The net normal force that results from those two components depends on the available secondary. A secondary with only aluminium produces a repulsive normal force, while a secondary with steel and aluminium plates produces a net attractive force, and a secondary with only a steel plate produces a relatively larger attractive normal force. That attractive normal force is employed for keeping the primary magnetically attracted to the surface of secondary, when available.

## 3. Electromagnetic Field Distribution Equations

An analytical 2D model was developed in rectangular co-ordinates for predicting the distribution of the electromagnetic field based on [5,6,7,8,9,10]. In the analysis, the contribution of the *x*-winding to the production of force in the *x*-direction is considered. In the analytical model, the induction planar actuator was divided into layers. Two analytical models for foreseeing the behaviour of the fields were developed: (a) considering the surface under inspection formed by two layers of different materials: aluminium and a ferromagnetic material (double-layered secondary); (b) the surface under study has one layer of a conducting material (single-layered secondary).

### 3.1. Equations for the Double-Layered Secondary

**Figure 3:**
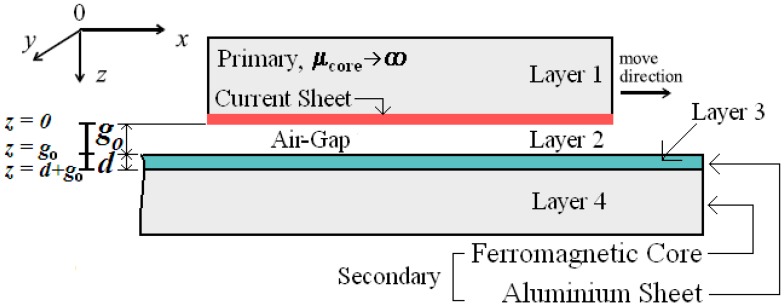
The layer model of the induction planar actuator.

A longitudinal section of the analytical model of the induction planar actuator is shown in Figure 3, considering that the secondary is formed by aluminium and ferromagnetic slabs. The slotted ferromagnetic core of the primary corresponds to layer 1. Each phase of the primary was represented by an infinitely thin sheet of current with a linear current density, $J_{m}$, in A/m.

The air-gap corresponds to the layer 2. According to what is described previously, the secondary consists of two layers, an aluminium slab, layer 3, and a steel ferromagnetic core, layer 4, having the latter a field-dependent permeability $\mu_{fe}$. In this way, the actuator is modelled by four layers, and layer 1 is represented by $J_{m}$ [5].

The expression for $J_{m}$ was developed based on the constructive characteristics of the core and the windings of the primary. Each phase of the *x*-winding of the planar actuator under study is represented by a Fourier series, according to:

(1) $$J_{m}=\frac{4NI}{\pi l_{d}}\sum_{n=1,3..}\frac{1}{n}\left[\cos\left(\frac{n\pi }{l_{t}}\left(\frac{l_{f}}{2}+\frac{l_{d}}{K_{d}}\right)\right)-\cos\left(\frac{n\pi l_{f}}{2l_{t}}\right)\right]\cos\left(n\left(\frac{\pi x}{l_{t}}-\varphi_{f}\right)-\frac{\pi }{2}\right)\cos\left(\omega t+\varphi_{t}\right)$$

Using complex notation, after trigonometric transformation, the linear density current can be expressed by a sum of two travelling waves. The MMF produced by 3-phase currents in the primary can be described as a sum of two waves of MMF, $\exp j\left(\omega t+\varphi_{t}-n\left(\frac{\pi x}{l_{t}}-\varphi_{f}\right)+\frac{\pi }{2}\right)$ travels in the direction of the *x* coordinate (forward-travelling magnetic field), and the other wave, given by $\exp j\left(\omega t+\varphi_{t}+n\left(\frac{\pi x}{l_{t}}-\varphi_{f}\right)-\frac{\pi }{2}\right)$, travels in the opposite direction of the *x* coordinate (backward-travelling magnetic field) [10]. Making *p* = 1 and *p* = −1 [5], $J_{m}$ can be expressed in a short form as:

(2) $$J_{m}=\frac{2NI}{\pi l_{d}}\sum_{n=1,3..}\sum_{p}\frac{1}{n}\left[\left[\cos\left(\frac{n\pi }{l_{t}}\left(\frac{l_{f}}{2}+\frac{l_{d}}{K_{d}}\right)\right)-\cos\left(\frac{n\pi l_{f}}{2l_{t}}\right)\right]\cdot \left[\exp j\left(\omega t+\varphi_{t}-pn\left(\frac{\pi x}{l_{t}}-\varphi_{f}\right)+p\frac{\pi }{2}\right)\right]\right]$$

In Equation (1), N is the number of turns of each coil, I is the current peak value in each coil, $l_{d}$ is the slot opening, $l_{f}$ is the tooth tip width (by the air-gap), $\varphi_{f}$ is the shift of the slot centre in relation to the coordinate system, $\varphi_{t}$ is the phase displacement angle, $l_{t}$ is the pole pitch, *n* is the number of the space harmonic, with only the odd ones being relevant, ω=2π f, and f is the frequency of currents in the primary winding. The coefficient $K_{d}$ is a factor that corrects the shape of the distribution of the magnetic flux density through the x-axis. It is described in Section 3.4. Equation (2) is represented in a short form by using *p* in the exponential function that represents the time–spatial variation of $J_{m}$. When *p* is equal to 1, the exponential function relates to the MMF wave that travels in the positive direction of the *x* coordinate; when *p* is equal to −1, the exponential function relates to a wave of MMF that travels in the opposite direction of x. 

The Maxwell’s equations that govern the electromagnetic behaviour of the actuator under study are $\nabla \times \vec{H}=\vec{J}$, $\nabla \cdot \vec{B}=0$ and $\nabla \times \vec{E}=-\frac{\partial \vec{B}}{\partial t}$ [5], where $\vec{H}$ is the magnetic field vector, $\vec{J}$ the current density vector in A/m^2^, $\vec{B}$ the magnetic flux density vector, $\vec{E}$, the electric field intensity vector and t is the time. Based on these equations and considering that in a moving medium, $\vec{J}=\sigma \left(\vec{E}+\left(\vec{v}\times \vec{B}\right)\right)$, with σ being equal to the conductivity of a layer and μ as its magnetic permeability, it is possible to obtain the equation that describes the behaviour of the magnetic vector potential, $\vec{A}$, inside layers 2, 3, and 4. Knowing that $\vec{B}$ is related to $\vec{A}$ by $\vec{B}=\nabla \times \vec{A}$, one can obtain [5]:

(3) $$\vec{J}=\nabla \times \frac{\nabla \times \vec{A}}{\mu }$$

and:

(4) $$\vec{E}=-\frac{\partial \vec{A}}{\partial t}$$

Applying Equations (3) and (4) according to the Ohm’s law for a moving medium, it results in:

(5) $$\nabla \times \frac{\nabla \times \vec{A}}{\mu }=\sigma \left(-\frac{\partial \vec{A}}{\partial t}+\left(\vec{v}\times \left(\nabla \times \vec{A}\right)\right)\right)$$

where $\vec{v}$ is the relative speed of the primary with respect to the secondary, here assumed in the *x*-direction, so $\vec{v}=v_{x}{\vec{a}}_{x}$. In the *x*-coils, the currents flow in the *y*-direction only and that implies that $\vec{J}=J_{y}{\vec{a}}_{y}$ and $\vec{A}=A_{y}{\vec{a}}_{y}$. Since the generation of the magnetic vector potential, $\vec{A}$, is produced by the current in the coils of the primary, $\vec{A}$ can also be expressed by a sum of two waves of magnetic potential vector according to:

(6) $$\vec{A}(x,z,t)=\sum_{n}\left[\begin{array}{l} A_{y}^{+}(z)\exp j\left(\omega t+\varphi_{t}-n\left(\frac{\pi x}{l_{t}}-\varphi_{f}\right)+\frac{\pi }{2}\right)\\ +A_{y}^{-}(z)\exp j\left(\omega t+\varphi_{t}+n\left(\frac{\pi x}{l_{t}}-\varphi_{f}\right)-\frac{\pi }{2}\right) \end{array}\right]{\vec{a}}_{y}=A_{y}{\vec{a}}_{y}={\vec{A}}_{}^{+}+{\vec{A}}_{}^{-}$$

In Equation (6), $A_{y}^{+}\left(z\right)$ and $A_{y}^{-}\left(z\right)$, where the superscript + refers to the forward-travelling field and - to the backward-travelling field, must be determined [12]. The speed of the primary can be represented as a function of the slip *s* as $v_{x}=\left(1-s\right)v_{s_{x}}$, and $v_{s_{x}}$ is the speed of the travelling magnetic field produced by the currents in the *x*-winding. The product of $v_{s_{x}}$ by $\pi /l_{t}$ results in ω. The corresponding slips to each harmonic component, $s_{n}^{+}$ and $s_{n}^{-}$, are represented by $s_{n}^{+}=1-n(1-s)$ and $s_{n}^{-}=1+n\left(1-s\right)$. Based on these considerations, when the solution of Equation (5) takes into account each wave separately, after some operations, it results in:

(7) $$-{\left(\frac{n\pi }{l_{t}}\right)}^{2}A_{y}^{+}(z)+\frac{\partial^{2}A_{y}^{+}(z)}{\partial z^{2}}=j\mu \sigma \omega s_{n}^{+}A_{y}^{+}(z)\text{ and }-{\left(\frac{n\pi }{l_{t}}\right)}^{2}A_{y}^{-}(z)+\frac{\partial^{2}A_{y}^{-}(z)}{\partial z^{2}}=j\mu \sigma \omega s_{n}^{-}A_{y}^{-}(z)$$

After some operations, the solution to Equation (7) is given by Equation (8) [12]. The total potential is the sum of the solution for all values of *n*:

(8) $$A_{y_{i}}^{+}(z)=\left[C_{i}^{+}\exp(\alpha_{i}^{+}z)+D_{i}^{+}\exp(-\alpha_{i}^{+}z)\right]\text{ and }A_{y_{i}}^{-}(z)=\left[C_{i}^{-}\exp(\alpha_{i}^{-}z)+D_{i}^{-}\exp(-\alpha_{i}^{-}z)\right]$$

In Equation (8), the subscript *i* represents the layer number, *i.e.*, 2, 3 and 4 for the air-gap, aluminium and secondary iron core, respectively, and $C_{i}^{+}$, $D_{i}^{+}$, $C_{i}^{-}$ and $D_{i}^{-}$ must be obtained. For the secondary, $\alpha_{i}^{+}$ and $\alpha_{i}^{-}$ are expressed by [12]:

(9) $$\alpha_{i}^{+}=\sqrt{\beta^{2}+\gamma_{i}^{+}{}_{}^{2}}=\sqrt{{\left(\frac{n\pi }{l_{t}}\right)}^{2}+j\mu_{i}\sigma_{i}\omega s_{n}^{+}}\text{ and }\alpha_{i}^{-}=\sqrt{\beta^{2}+\gamma_{i}^{-}{}_{}^{2}}=\sqrt{{\left(\frac{n\pi }{l_{t}}\right)}^{2}+j\mu_{i}\sigma_{i}\omega s_{n}^{-}}$$

The constants $\gamma_{i}^{+}$ and $\gamma_{i}^{-}$ are obtained through:

(10) $$\gamma_{i}^{+}=\sqrt{j\mu_{i}\sigma_{i}\omega s_{n}^{+}}\text{ and }\gamma_{i}^{-}=\sqrt{j\mu_{i}\sigma_{i}\omega s_{n}^{-}}$$

Boundary conditions related to the components of the magnetic field were imposed at the interfaces between layers. They relate the *x*-components of the magnetic field and the *z*-components of the magnetic flux density of two layers. They allow the solution of Equation (7) for each layer and lead to the electromagnetic field equations. The conditions are listed as:

*i*. $H_{x_{2}}=J_{m}$ in z=0
*ii*. $H_{x_{2}}=H_{{}_{x_{3}}}$ and $B_{z_{2}}=B_{z_{3}}$ in z=g
*iii*. $H_{x_{3}}=H_{{}_{x4}}$ and $B_{z_{3}}=B_{z_{4}}$ in z=g+d
*iv*. ${\vec{B}}_{{}_{4}}=0$, when z=∞

where *g* is the air-gap length corrected by multiplying *g_o_* by the Carter’s coefficient, according to what is described in Section 3.4. The *x* and *z*-components of the magnetic flux density are equal to $B_{x_{i}}^{}=-\frac{\partial A_{y_{i}}^{}}{\partial z}$ and $B_{z_{i}}^{}=\frac{\partial A_{y_{i}}^{}}{\partial x}$, respectively. For each one of the waves in Equation (8), conditions from *i* to *iv* form a set of six equations and six unknowns. The solution of the equations set leads to obtaining of $C_{2}^{}$, $D_{2}$, $C_{3}^{}$, $D_{3}$, $C_{4}^{}$ and $D_{4}$. The *x* and *z*-components of the magnetic flux density vector and the *y*-component of the electric field intensity vector produced by *x*-winding inside the layers are given by the following set of equations:

(11) $$B_{x_{2}}^{}(x,z,t)=\sum_{nf}^{}\sum_{n}^{}\sum_{p}^{}\left(-\frac{\mu_{0}J_{c}\alpha_{2}}{M}\left[\begin{array}{l} \left[\sinh(\alpha_{2}(z-g))\left(D\cosh(\alpha_{3}d)+\sinh(\alpha_{3}d)\right)\right]\\ -\left[\cosh(\alpha_{2}(z-g))\left(B\cosh(\alpha_{3}d)+BD\sinh(\alpha_{3}d)\right)\right] \end{array}\right]\exp\left(j(\omega \;t-p\beta x)\right)\right)$$

(12) $$B_{z_{2}}^{}(x,z,t)=\sum_{nf}^{}\sum_{n}^{}\sum_{p}^{}j\left(-\frac{\mu_{0}J_{c}p\beta }{M}\left[\begin{array}{l} \left[\cosh(\alpha_{2}(z-g))\left(D\cosh(\alpha_{3}d)+\sinh(\alpha_{3}d)\right)\right]\\ -\left[\sinh(\alpha_{2}(z-g))\left(B\cosh(\alpha_{3}d)+BD\sinh(\alpha_{3}d)\right)\right] \end{array}\right]\exp\left(j(\omega \;t-p\beta x)\right)\right)$$

(13) $$E_{y_{2}}^{}(x,z,t)=\sum_{nf}^{}\sum_{n}^{}\sum_{p}^{}j\left(-\frac{\mu_{0}J_{c}\omega }{M}\left[\begin{array}{l} \left[\cosh(\alpha_{2}(z-g))\left(D\cosh(\alpha_{3}d)+\sinh(\alpha_{3}d)\right)\right]\\ -\left[\sinh(\alpha_{2}(z-g))\left(B\cosh(\alpha_{3}d)+BD\sinh(\alpha_{3}d)\right)\right] \end{array}\right]\exp\left(j(\omega \;t-p\beta x)\right)\right)$$

(14) $$B_{x_{3}}^{}(x,z,t)=\sum_{nf}^{}\sum_{n}^{}\sum_{p}^{}\left(-\frac{\mu_{0}J_{c}\alpha_{3}}{M}[D\sinh(\alpha_{3}(z-g-d))-\cosh(\alpha_{3}(z-g-d))]\exp\left(j(\omega \;s_{n}^{\pm }t-p\beta x\right)\right)$$

(15) $$B_{z_{3}}^{}(x,z,t)=\sum_{nf}^{}\sum_{n}^{}\sum_{p}^{}j\left(-\frac{\mu_{0}J_{c}p\beta }{M}[D\cosh(\alpha_{3}(z-g-d))-\sinh(\alpha_{3}(z-g-d))]\exp\left(j(\omega \;s_{n}^{\pm }t-p\beta x)\right)\right)$$

(16) $$E_{y_{3}}^{}(x,z,t)=\sum_{nf}^{}\sum_{n}^{}\sum_{p}^{}j\left(-\frac{\mu_{0}J_{c}\omega \;s_{n}^{\pm }}{M}[D\cosh(\alpha_{3}(z-g-d))-\sinh(\alpha_{3}(z-g-d))]\exp\left(j(\omega \;s_{n}^{\pm }t-p\beta x)\right)\right)$$

(17) $$B_{x_{4}}^{}(x,z,t)=\sum_{nf}^{}\sum_{n}^{}\sum_{p}^{}\left(\frac{\mu_{0}J_{c}\alpha_{4}}{M}D\exp(-\alpha_{4}(z-g-d))\exp\left(j(\omega \;s_{n}^{\pm }t-p\beta x)\right)\right)$$

(18) $$B_{z_{4}}^{}(x,z,t)=\sum_{nf}^{}\sum_{n}^{}\sum_{p}^{}j\left(-\frac{\mu_{0}J_{c}p\beta }{M}D\exp(-\alpha_{4}(z-g-d))\exp\left(j(\omega \;s_{n}^{\pm }t-p\beta x)\right)\right)$$

(19) $$E_{y_{4}}^{}(x,z,t)=\sum_{nf}^{}\sum_{n}^{}\sum_{p}^{}j\left(-\frac{\mu_{0}J_{c}\omega \;s_{n}^{\pm }}{M}D\exp(-\alpha_{4}(z-g-d))\exp\left(j(\omega \;s_{n}^{\pm }t-p\beta x)\right)\right)$$

The time–spatial variation of all electric and magnetic fields in the secondary is expressed by $\exp\left(j(\omega \;s_{n}^{+}t-\beta x)\right)$ for a forward-travelling magnetic field and by $\exp\left(j(\omega \;s_{n}^{-}t+\beta x)\right)$ for a backward-travelling magnetic field since the primary moves with speed $v_{s_{x}}$, so ω is multiplied by the slip $s_{n}^{\pm }=1\mp n\left(1-s\right)=1-np\left(1-s\right)$ because those equations are represented in the coordinate system related to the secondary. In Equations (11) to (19), $D=\mu_{4}\;\alpha_{3}/\mu_{3}\;\alpha_{4}$, $B=\mu_{2}\alpha_{3}/\mu_{3}\alpha_{2}$, 2, 3 and 4 are the layer number of air, aluminium and secondary iron core, respectively, and *d* is the aluminium length through *z*-axis, *p* = −1, 1, *nf* is the number of phases, *M* is given by Equation (20) and $J_{c}$ by Equation (21):

(20) $$M=\alpha_{2}\left[\left[\cosh(\alpha_{2}g)(B\cosh(\alpha_{3}d)+DB\sinh(\alpha_{3}d))\right]+\left[\sinh(\alpha_{2}g)(D\cosh(\alpha_{3}d)+\sinh(\alpha_{3}d))\right]\right]$$

(21) $$J_{c}=\frac{2NI}{n\pi l_{d}}\left[\cos\left(\frac{n\pi }{l_{t}}\left(\frac{l_{f}}{2}+\frac{l_{d}}{K_{d}}\right)\right)-\cos\left(\frac{n\pi l_{f}}{2l_{t}}\right)\right]\left[\exp j\left(\varphi_{t}+(pn\varphi_{f})+p\frac{\pi }{2}\right)\right]$$

### 3.2. Equations for the Single-Layered Secondary

In the development of the analytical model for a secondary with only one conductive plate, the slotted ferromagnetic core of the primary corresponds to layer 1 and the air-gap to layer 2. Layer 3 represents the conductive plate and $M=\alpha_{2}\left[B\cosh\left(\alpha_{2}g\right)+\sinh\left(\alpha_{2}g\right)\right]$. Now, the actuator is modelled by three layers. Following the same steps of the Section 3.1, one can obtain the *x* and *z*-components of the magnetic flux density vector and the *y*-component of the electric field intesity vector produced by *x*-winding inside the layers by using the set of equations as follows:

(22) $$B_{x_{2}}^{}(x,z,t)=\sum_{nf}^{}\sum_{n}^{}\sum_{p}^{}\left(-\frac{\mu_{0}J_{c}\alpha_{2}}{M}\left[-B\cosh(\alpha_{2}(z-g))+\sinh(\alpha_{2}(z-g))\right]\exp\left(j(\omega \;t-p\beta x)\right)\right)$$

(23) $$B_{z_{2}}^{}(x,z,t)=\sum_{nf}^{}\sum_{n}^{}\sum_{p}^{}j\left(-\frac{\mu_{0}J_{c}p\beta }{M}\left[\cosh(\alpha_{2}(z-g))-B\sinh(\alpha_{2}(z-g))\right]\exp\left(j(\omega \;t-p\beta x)\right)\right)$$

(24) $$E_{y_{2}}^{}(x,z,t)=\sum_{nf}^{}\sum_{n}^{}\sum_{p}^{}j\left(-\frac{\mu_{0}J_{c}\omega }{M}\left[\cosh(\alpha_{2}(z-g))-B\sinh(\alpha_{2}(z-g))\right]\exp\left(j(\omega \;t-p\beta x)\right)\right)$$

(25) $$B_{x_{3}}^{}(x,z,t)=\sum_{nf}^{}\sum_{n}^{}\sum_{p}^{}\left(\frac{\mu_{0}J_{c}\alpha_{3}}{M}\exp(-\alpha_{3}(z-g))\exp\left(j(\omega \;s_{n}^{\pm }t-p\beta x)\right)\right)$$

(26) $$B_{z_{3}}^{}(x,z,t)=\sum_{nf}^{}\sum_{n}^{}\sum_{p}^{}j\left(-\frac{\mu_{0}J_{c}p\beta }{M}\exp(-\alpha_{3}(z-g))\exp\left(j(\omega \;s_{n}^{\pm }t-p\beta x)\right)\right)$$

(27) $$E_{y_{3}}^{}(x,z,t)=\sum_{nf}^{}\sum_{n}^{}\sum_{p}^{}j\left(-\frac{\mu_{0}J_{c}\omega s_{n}^{\pm }}{M}\exp(-\alpha_{3}(z-g))\exp\left(j(\omega \;s_{n}^{\pm }t-p\beta x)\right)\right)$$

### 3.3. 3D Analytical Model

When both windings, x and y, are taken into account, an analytical 3D model was employed in order to analyse the behaviour of the forces produced by the actuator. The 3D model was deduced based on the 2D model presented is this paper. The dimensions of the magnetic circuit, the windings and phase currents are symmetrical. The current densities are presented in Equations (28) and (29). They describe the behaviour of the sheet of current produced by the x-winding, $J_{my}$, and by the y-winding, $J_{mx}$. Both are expressed as a sum of four travelling waves:

(28) $$J_{my}=\frac{NI}{\pi l_{d}}\sum_{n=1,3..}\sum_{p}\sum_{m=0,1,2.}\sum_{q}\left[\begin{array}{l} \frac{1}{n}\left[\cos\left(\frac{n\pi }{l_{t}}\left(\frac{l_{f}}{2}+\frac{l_{d}}{K_{d}}\right)\right)-\cos\left(\frac{n\pi l_{f}}{2l_{t}}\right)\right]J_{mym}\\ \cdot \left[\exp j\left(\omega t+\varphi_{tx}-pn\left(\frac{\pi x}{l_{t}}-\varphi_{fx}\right)+p\frac{\pi }{2}-qm\left(\frac{2\pi y}{l_{p}}\right)\right)\right] \end{array}\right]$$

(29) $$J_{mx}=\frac{NI}{\pi l_{d}}\sum_{n=1,3..}\sum_{p}\sum_{m=0,1,2.}\sum_{q}\left[\begin{array}{l} \frac{1}{n}\left[\cos\left(\frac{n\pi }{l_{t}}\left(\frac{l_{f}}{2}+\frac{l_{d}}{K_{d}}\right)\right)-\cos\left(\frac{n\pi l_{f}}{2l_{t}}\right)\right]J_{mxm}\\ \cdot \left[\exp j\left(\omega t+\varphi_{ty}-pn\left(\frac{\pi y}{l_{t}}-\varphi_{fy}\right)+p\frac{\pi }{2}-qm\left(\frac{2\pi x}{l_{p}}\right)\right)\right] \end{array}\right]$$

where:

(30) $$J_{mym}=J_{mxm}=\frac{l_{f}}{l_{p}}\text{ at }m=0$$

(31) $$J_{mym}=J_{mxm}=\frac{2}{m\pi }\sin\left(\frac{m\pi l_{f}}{l_{p}}\right)\text{ when }m>0$$

In Equations (28) and (29), $\varphi_{fx}$ and $\varphi_{fy}$ are the shift of the slot centre of the *x* and *y*-winding in relation to the *x* and *y*-coordinate, $\varphi_{tx}$ and $\varphi_{ty}$ are the phase displacement angle of the *x* and *y*-winding, *n* and *m* are the number of the space harmonics, $l_{p}$ is the tooth pitch. Similar to *p, q* is employed on the sum of the *m* harmonics and equals to 1 and -1. As *p*, it allows representing the exponential function that gives the time–spatial variation of $J_{mx}$ and $J_{my}$ in a short form. The magnetic potential vector, $\vec{A}$, has *x* and *y*-components, and it is expressed by $\bar{A}\left(x,y,z,t\right)=A_{x}{\bar{a}}_{x}+A_{y}{\bar{a}}_{y}$. The generation of $A_{x}$ and $A_{y}$ is made by $J_{mx}$ and $J_{my}$, respectively. So, each component of $\bar{A}\left(x,y,z,t\right)$ can be expressed as a sum of four travelling waves. 

Following similar steps of the 2D analytical model, after some operations, the solution to the equations of the components of the magnetic potential vector and the boundary conditions relating to the components of the magnetic field allow obtaining the components of the magnetic flux density vector and of the electric field intensity vector in all layers. One of the results of the analysis is the components of the resulting magnetic flux density vector in the air gap for the double-layered secondary:

(32) $$B_{x_{2_{R}}}^{}(x,y,z,t)=\sum_{nf}^{}\sum_{n}^{}\sum_{p}^{}\sum_{m}\sum_{q}\left(\begin{array}{l} -\frac{\mu_{0}J_{yc}\alpha_{x2}}{M}\left[\begin{array}{l} \left[\sinh(\alpha_{x2}(z-g))\left(D\cosh(\alpha_{x3}d)+\sinh(\alpha_{x3}d)\right)\right]\\ -\left[\cosh(\alpha_{x2}(z-g))\left(B\cosh(\alpha_{x3}d)+BD\sinh(\alpha_{x3}d)\right)\right] \end{array}\right]\\ \exp\left(j(\omega \;t-p\beta x-qm\left(\frac{2\pi y}{l_{p}}\right)\right) \end{array}\right)$$

(33) $$B_{y_{2_{R}}}^{}(x,y,z,t)=\sum_{nf}^{}\sum_{n}^{}\sum_{p}\sum_{m}\sum_{q}^{}\left(\begin{array}{l} \frac{\mu_{0}J_{xc}\alpha_{y2}}{M}\left[\begin{array}{l} \left[\sinh(\alpha_{y2}(z-g))\left(D\cosh(\alpha_{y3}d)+\sinh(\alpha_{y3}d)\right)\right]\\ -\left[\cosh(\alpha_{y2}(z-g))\left(B\cosh(\alpha_{y3}d)+BD\sinh(\alpha_{y3}d)\right)\right] \end{array}\right]\\ \exp\left(j(\omega \;t-p\beta y-qm\left(\frac{2\pi x}{l_{p}}\right)\right) \end{array}\right)$$

(34) $$\begin{array}{r} B_{z_{2}{}_{R}}^{}(x,y,z,t)=\sum_{nf}^{}\sum_{n}^{}\sum_{p}^{}\sum_{m}\sum_{q}j[\left[\begin{array}{l} -\frac{\mu_{0}J_{yc}p\beta }{M}\left[\begin{array}{l} \left[\cosh(\alpha_{x2}(z-g))\left(D\cosh(\alpha_{x3}d)+\sinh(\alpha_{x3}d)\right)\right]\\ -\left[\sinh(\alpha_{x2}(z-g))\left(B\cosh(\alpha_{x3}d)+BD\sinh(\alpha_{x3}d)\right)\right] \end{array}\right]\\ \exp\left(j(\omega \;t-p\beta x-qm\left(\frac{2\pi y}{l_{p}}\right)\right) \end{array}\right]\\ +\left[\begin{array}{l} \frac{\mu_{0}J_{xc}p\beta }{M}\left[\begin{array}{l} \left[\cosh(\alpha_{y2}(z-g))\left(D\cosh(\alpha_{y3}d)+\sinh(\alpha_{y3}d)\right)\right]\\ -\left[\sinh(\alpha_{y2}(z-g))\left(B\cosh(\alpha_{y3}d)+BD\sinh(\alpha_{y3}d)\right)\right] \end{array}\right]\\ \exp\left(j(\omega \;t-p\beta y-qm\left(\frac{2\pi x}{l_{p}}\right)\right) \end{array}\right]] \end{array}$$

where $J_{xc}$ and $J_{yc}$ are given by:

(35) $$J_{xc}=\frac{NI}{\pi l_{d}n}\left[\cos\left(\frac{n\pi }{l_{t}}\left(\frac{l_{f}}{2}+\frac{l_{d}}{K_{d}}\right)\right)-\cos\left(\frac{n\pi l_{f}}{2l_{t}}\right)\right]J_{mxm}\cdot \left[\exp j\left(\varphi_{ty}+pn\varphi_{fy}+p\frac{\pi }{2}\right)\right]$$

(36) $$J_{yc}=\frac{NI}{\pi l_{d}n}\left[\cos\left(\frac{n\pi }{l_{t}}\left(\frac{l_{f}}{2}+\frac{l_{d}}{K_{d}}\right)\right)-\cos\left(\frac{n\pi l_{f}}{2l_{t}}\right)\right]J_{mym}\cdot \left[\exp j\left(\varphi_{tx}+pn\varphi_{fx}+p\frac{\pi }{2}\right)\right]$$

The speed of the primary is equal to the vector sum of $v_{x}$ and $v_{y}$, respectively the components of primary velocities when the *x* and *y*-windings are fed by current. When the *x*-winding is fed by current, the velocity of the primary $v_{x}$ is equal to $(1-s_{x})v_{s_{x}}$. The corresponding slips for each harmonic component, $s_{xn}^{+}$ and $s_{xn}^{-}$, are represented by $s_{xn}^{+}=1-n(1-s_{x})$ and $s_{xn}^{-}=1+n(1-s_{x})$. Regarding to the *y*-winding, $v_{y}=(1-s_{y})v_{s_{y}}$, where $v_{s_{y}}$ is the speed of the travelling magnetic field produced by the currents in the same winding and the slips to each harmonic component, $s_{yn}^{+}$ and $s_{yn}^{-}$, are represented by $s_{yn}^{+}=1-n(1-s_{y})$ and $s_{yn}^{-}=1+n(1-s_{y})$.

### 3.4. Corrections Factors Applied to the Electric and Magnetic Field Equations

In this work, the air-gap is corrected by multiplying its length, *g_o_*, by the Carter’s coefficient, $K_{C}$. This coefficient takes into account the stator slotting, and for a slotless secondary it is expressed by [12] as:

(37) $$K_{C}=\frac{l_{f}+l_{d}}{\left(l_{f}+l_{d}\right)-\eta_{1}g_{m}}$$

where $g_{m}=g_{o}+d$ is the magnetic air-gap for case 1 and $\eta_{1}$ is given by:

(38) $$\eta_{1}=\frac{4}{\pi }\left[\frac{l_{d}}{2g_{m}}\arctan\left(\frac{l_{d}}{2g_{m}}\right)-\ln\sqrt{\left(1+{\left(\frac{l_{d}}{2g_{m}}\right)}^{2}\right)}\right]$$

The authors of [13] present a modified equation for correcting the stator slotting when the secondary is slotless, according to:

(39) $$K_{C}=\frac{l_{f}+l_{d}}{l_{f}+\left(\frac{4g_{o}}{\pi }\ln\left(1+\frac{\pi l_{d}}{4g_{o}}\right)\right)}$$

The equivalent air-gap length in a 2D analytical model is expressed by:

(40) $$g=K_{C}g_{o}$$

In the 3D model, the equivalent air-gap length is equal to $g=K_{xC}K_{yC}g_{o}$ and $K_{xC}$ and $K_{yC}$ are calculated by using the dimensions of $l_{f}$ and $l_{d}$ throughout the *x* and *y*-axes.

The relative magnetic permeability of the ferromagnetic material of the secondary is corrected to account for the effects of hysteresis and saturation by means of a relative equivalent magnetic permeability, $\mu_{re}$, according to the expression [12]:

(41) $$\mu_{re}=\mu_{r}\left(\mu^{\prime }-j\mu^{\prime \prime }\right)$$

where $\mu_{r}$ is the relative magnetic permeability of the ferromagnetic material of the secondary. The real and imaginary permeabilities, $\mu^{\prime }$ and $\mu^{\prime \prime }$ , respectively, are obtained according the following development based on [12]. For the ferromagnetic plate, the constants are obtained by:

(42) $$\gamma_{fe}^{+}=\sqrt{j\mu_{re}\mu_{0}\sigma_{fe}\omega s_{n}^{+}}\text{ and }\gamma_{fe}^{-}=\sqrt{j\mu_{re}\mu_{0}\sigma_{fe}\omega s_{n}^{-}}$$

or:

(43) $$\begin{array}{cc} \gamma_{fe}^{\pm } & =\sqrt{j\mu_{re}\mu_{0}\sigma_{fe}\omega s_{n}^{\pm }}=\sqrt{j\mu_{re}\mu_{0}\sigma_{fe}\omega (1\mp n(1-s))}=\sqrt{j\mu_{0}\mu_{r}\left(\mu^{\prime }-j\mu^{\prime \prime }\right)\sigma_{fe}\omega (1\mp n(1-s))}\\ & =\sqrt{j2\pi f\mu_{0}\mu_{r}\left(\mu^{\prime }-j\mu^{\prime \prime }\right)\sigma_{fe}\left(1\mp n\left(1-s\right)\right)}=\sqrt{{\left(a_{R}^{}+ja_{x}^{}\right)}^{2}\pi f\mu_{0}\mu_{r}\sigma_{fe}(1\mp n(1-s))} \end{array}$$

and:

(44) $$j2\left(\mu^{\prime }-j\mu^{\prime \prime }\right)=\left(a_{R}^{2}+2ja_{R}^{}a_{x}^{}-a_{x}^{2}\right)$$

(45) $$\mu^{\prime }=a_{R}a_{x}\text{ and }\mu^{\prime \prime }=0.5(a_{R}^{2}-a_{x}^{2})$$

In Equations (44) and (45), $a_{R}$ and $a_{x}$ are coefficients that depend on the magnetic field on the surface of the ferromagnetic material. The author of [14] describes a detailed investigation taking into account variations of $a_{R}$ and $a_{x}$ with the field intensity. 

According to what was described previously, $K_{d}$ is a factor that corrects the shape of the magnetic flux density through the *x*-axis. The winding of the primary is formed in such a way that each coil is mounted around one tooth. The magnetic flux produced by one coil is distributed along of the tooth pitch. The $K_{d}$ factor takes into account the behaviour the magnetic flux between two adjacent teeth and it is used to introduce the fringe effect in the equation of the linear current density, $J_{m}$. For a planar actuator with $l_{f}$ > $l_{d}$, $K_{d}$ is given by:

(46) $$K_{d}=\frac{l_{f}+l_{d}}{l_{f}}$$

The application of this factor is proposed by the authors of this paper and it results from an empirical determination of the shape of the magnetic flux density in the air-gap. 

The transverse edge effect acts in the direction of stator slotting and provokes a decrease of the secondary conductivity [15]. In order to correct this effect, a coefficient is applied to the conductivity of the aluminium, according to:

(47) $$\sigma_{al}=K_{t}{\sigma^{\prime }}_{al}$$

where ${\sigma^{\prime }}_{al}$ is the actual conductivity of the aluminium. The coefficient $K_{t}$ or Russel and Norsworthy factor, $K_{RN}$, is calculated by [9] as:

(48) $$K_{RN}=1-\frac{\tanh\left(\pi \frac{L}{2l_{t}}\right)}{\left(\pi \frac{L}{2l_{t}}\right)\left[1+\tanh\left(\pi \frac{L}{2l_{t}}\right)\tanh\left(\pi \frac{\left(w_{d}-L\right)}{2l_{t}}\right)\right]}$$

In Equation (48), L and $w_{d}$ are the mover and the aluminium width through the *y*-axis, respectively, for the *x*-winding, and through *x*-axis for the *y*-winding. 

In order to correct the transverse edge effect in the ferromagnetic back iron, the factor $K_{s}$ is applied to the conductivity of the ferromagnetic material, according to $\sigma_{fe}={\sigma^{\prime }}_{fe}/K_{s}$, where ${\sigma^{\prime }}_{fe}$ is the actual conductivity of the ferromagnetic material. In [10], four different equations are presented for the correction of the transverse effect for a linear induction motor with a steel reaction plate. Here, the authors make use of one of those equations presented in [10], given by:

(49) $$K_{s}=1+\left(\frac{2l_{t}}{\pi w_{d}}\right)$$

### 3.5. Equations of Forces

The thrust and net normal forces per unit area produced by the *x*-winding were calculated by means of the Maxwell Tensor, according to:

$$f_{x}=-0.5\mu_{0}ℜe[H_{z}H_{x}^{*}]$$

(50) $$f_{y}=0.5\mu_{0}ℜe[H_{z}H_{y}^{*}]$$

$$f_{z}=0.25\mu_{0}ℜe[H_{z}H_{z}^{*}-H_{x}H_{x}^{*}-H_{y}H_{y}^{*}]$$

In Equation (50), $H_{x}$, $H_{y}$ and $H_{z}$ are the resulting *x, y* and *z*-components of the magnetic field vector expression in the air-gap at *z* = g and the superscript * denotes the complex conjugate. 

### 3.6. Electromagnetic Simulation

In order to validate the analytical model, the induction planar motor was fully modelled using Ansys Maxwell 16, a 3D field simulation package. Through the transient magnetic analysis capability of this package, the magnetic fields are computed at each time step set for the time domain of the simulation. The formulation of the solver is based on a current vector potential in solid conductors and a scalar potential over the entire field domain, and it allows the inclusion of nonlinear characteristics for the magnetic materials. To account for the effects of magnetic flux lines that pass through the air, the actuator was encircled in all directions by an air layer with the same size of the objects modelled, except for the simulation with only aluminium in the secondary, which required an air layer at least three times bigger under the aluminium plate to achieve steady results. Simulations were then carried out by considering the actuator windings star-connected and without a neutral connection. It was fed from a 60 Hz 3-phase source under the same conditions of current values obtained in experimental measurements. The simulations of the actuator were carried under static conditions, *i.e.*, at a slip of 1. They gave an assessment of the distribution of the magnetic flux in the air-gap, and the thrust and normal forces under the static condition. The model was formed from 317,385 tetrahedral elements. The simulations utilized a time step of 0.0008 s, which provided 20 steps per period of the excitation voltage. The simulation employed a time lapse of 10 periods, which was a sufficient time period to achieve a steady-state behaviour. 

**Figure 4:**
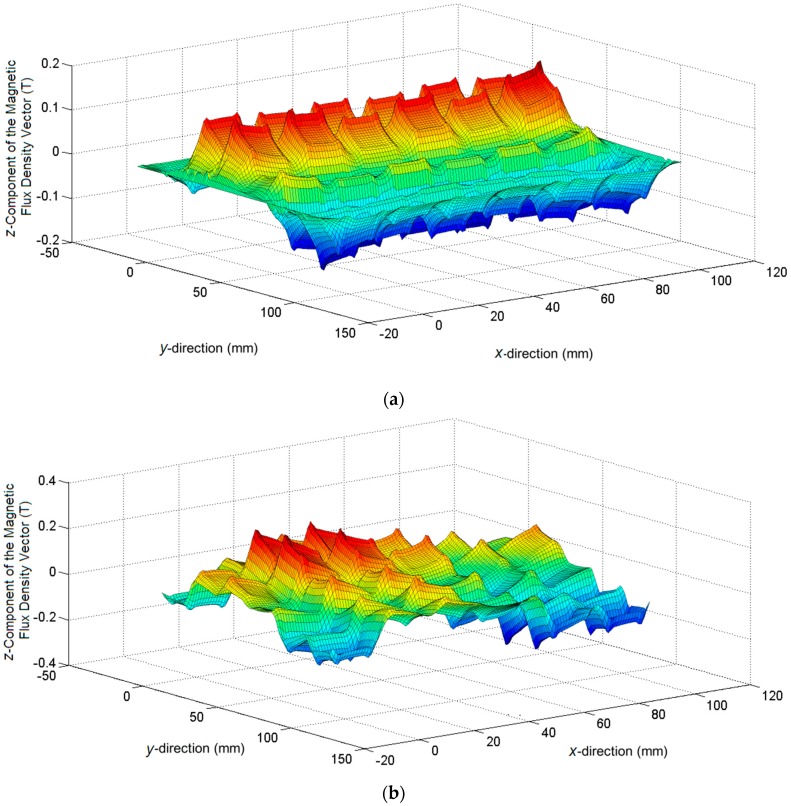
Surface plot of the normal component of the magnetic flux density in the air-gap, case 1: (**a**) with only *y*-winding fed with voltage; (**b**) with *x* and *y*-windings fed with voltage.

Figure 4 shows a surface plot of the *z*-axis component of the magnetic flux density in the middle of the air-gap of the device considering the case 1. The line voltage is equal to 60 V_rms_ (1.25 A of current in each coil) and only the *y*-axis winding is supplied in Figure 4a, while in Figure 4b, both *x* and *y*-windings are fed. It can be noted that there are peaks in the magnetic flux at the corners of each tooth, and that under some teeth in the same row, the flux density values are higher than others, and this pattern alternates. This is because coils closest to the air-gap have a larger contribution for the air-gap flux, and therefore a larger contribution to the force produced. The coils at the upper part of the teeth contribute less and have more leakage flux. From this figure, it is possible to verify the distortion of the air-gap magnetic field established by the currents in the primary, due to the eddy currents in the secondary. In addition, it also illustrates that it is necessary to alternate the coil location on the teeth in order ensure that the *x* and *y* axis characteristics are the same.

## 4. Measurement of Force

**Figure 5:**
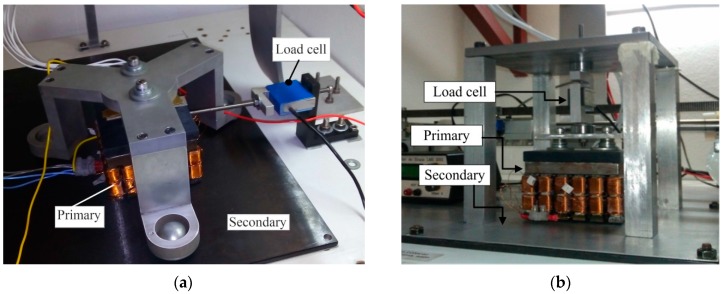
Experimental setup: (**a**) for thrust force measurement; (**b**) for normal force measurement.

A test rig was designed for the experimental part of the study. Measurements of the thrust and normal forces under static conditions (slip = 1) were taken using load cells. Figure 5a shows the test rig for measurement of thrust force. Figure 5b presents the test rig during the normal force measurements, with the actuator suspended by a load cell. The measurements were carried out first with only the *x*-axis winding excited by a 3-phase 60 Hz AC voltage. Then the same measurements of thrust and normal forces were taken with both windings excited. The measurements were carried out taking into account the three cases: (1) a double-layered secondary formed by aluminium and ferromagnetic materials; (2) a single aluminium layer and (3) a single ferromagnetic layer. In all three cases, the air-gap length was kept 2 mm large, the aluminium layer thickness equals to 1 mm and the ferromagnetic layer thickness is 5 mm.

## 5. Results 

**Figure 6:**
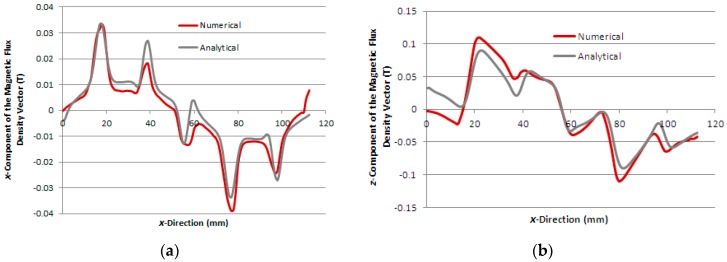
Components of the magnetic flux density vector as a function of *x*-direction, case 1: (**a**) *x*-component and (**b**) *z*-component.

**Figure 7:**
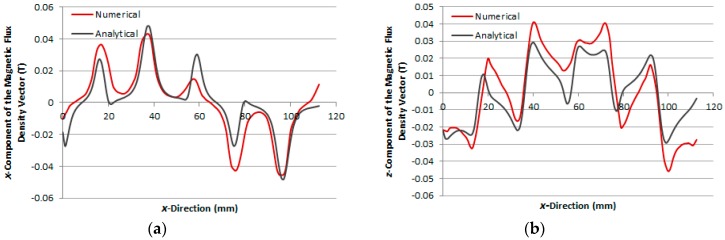
Components of the magnetic flux density vector as a function of *x*-direction, case 2: (**a**) *x*-component and (**b**) *z*-component.

**Figure 8:**
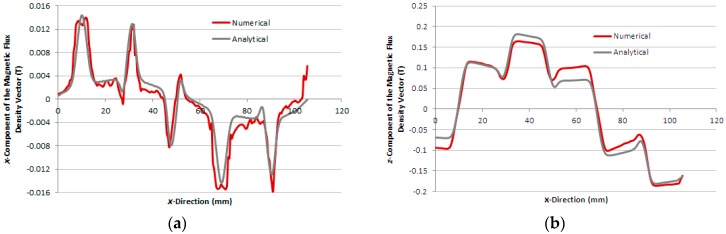
Components of the magnetic flux density vector as a function of *x*-direction, case 3: (**a**) *x*-component and (**b**) *z*-component.

Figure 6a,b show the graphs of the analytical and numerical *x* and *z*-component of the magnetic flux density vector, respectively, calculated in the boundary between the air-gap and the aluminium plate, case 1, when the *x*-winding is excited by a current of 2.0 A. Figure 7a,b present the same graphs for case 2 and, in Figure 8a,b, the same graphs for case 3, calculated in the air-gap at *z* = 1.7 mm, *i.e.*, 0.3 mm above the steel plate. 

**Figure 9:**
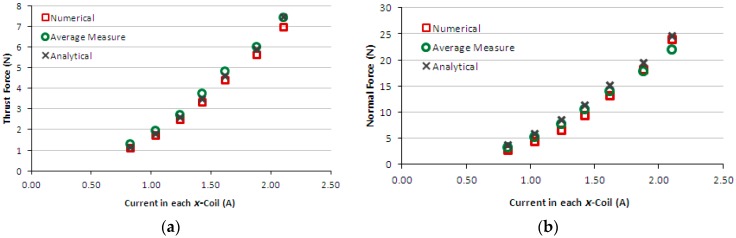
Case 1: (**a**) thrust force and (**b**) normal force produced by the induction planar actuator with only the *x*-winding excited.

**Figure 10:**
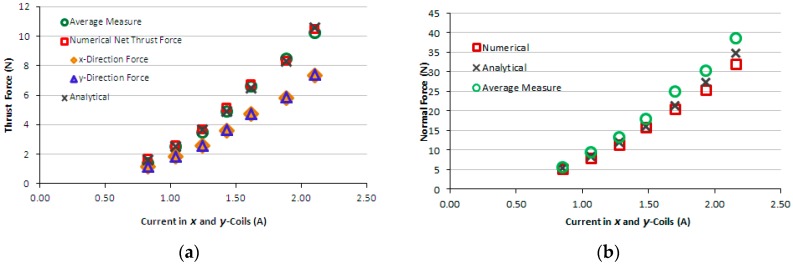
Case 1: (**a**) thrust force and (**b**) normal force produced by the induction planar actuator when both *x* and *y*-winding are under current.

Figure 9a,b present the graphs of thrust and normal measured forces produced by the induction planar actuator when only *x*-winding is fed, case 1. For the sake of comparison, numerical and analytical results are presented as well. Figure 10a,b present the graphs of thrust and normal results of forces produced by the induction planar actuator when both *x* and *y*-windings are fed, case 1. As seen in Figure 10a, the *x* and *y*-direction forces produced are equal, as expected, since there is symmetry involved, and those forces are slightly lower than the corresponding forces when only one winding is excited −1.50% lower on average. Additionally, a higher net thrust force is produced by the combination of the *x* and *y*-direction forces, according to Figure 10b. 

**Figure 11:**
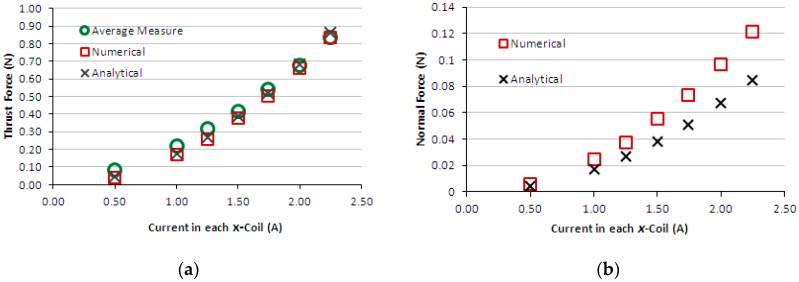
Case 2: (**a**) thrust force and (**b**) normal force produced by the induction planar actuator with only the *x*-winding excited.

**Figure 12:**
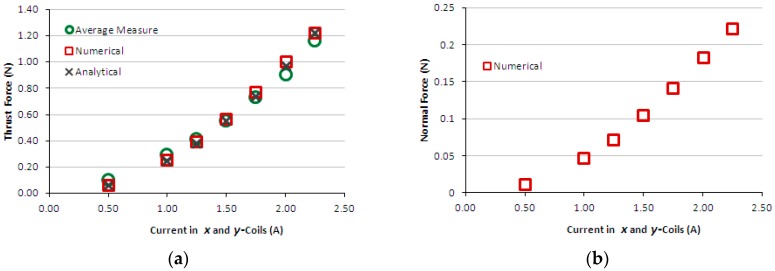
Case 2: (**a**) thrust force and (**b**) normal force produced by the induction planar actuator when both *x* and *y*-winding are under current.

**Figure 13:**
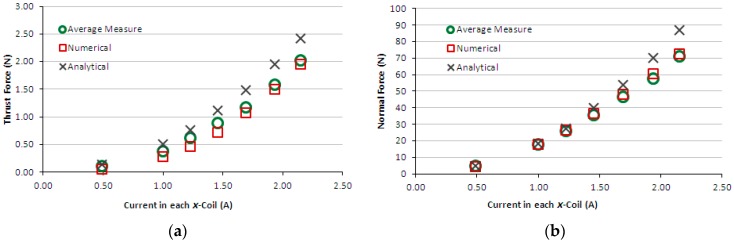
Case 3: (**a**) thrust force and (**b**) normal force produced by the induction planar actuator with only the *x*-winding excited.

**Figure 14:**
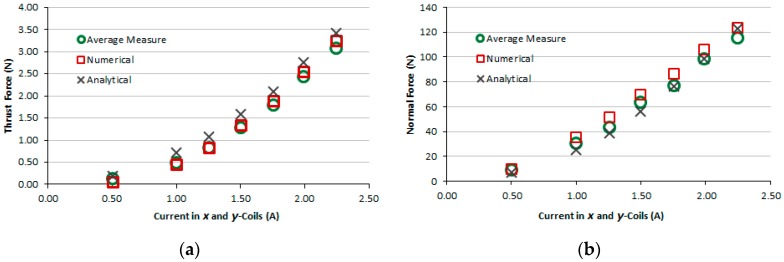
Case 3: (**a**) thrust force and (**b**) normal force produced by the induction planar actuator when both *x* and *y*-winding are under current.

Figure 11a presents the graph of thrust measured force produced by the induction planar actuator when only the *x*-winding is fed, case 2 – secondary with an aluminium plate. Again, for the sake of comparison, theoretical results are presented together. Figure 11b presents numerical and analytical results of normal levitation force for case 2. Figure 12a presents the graph of measured and theoretical thrust force produced by the induction planar actuator when both *x* and *y*-windings are fed in the same way, case 2. Figure 12b presents the graph of numerical normal force when both *x* and *y*-windings are under current, case 2. Figure 13a,b show similar graphs to Figure 9a,b for case 3-secondary formed by a plate of ferromagnetic material. Figure 14a,b present, for case 3, results of measured and theoretical thrust and normal forces when both *x* and *y*-windings are fed.

In all graphs, analytical calculations of the components of the magnetic flux density and forces were performed considering n and m harmonics up to the 499th order. For comparison and validation of the results, figures are obtained by the numerical and analytical models and by measurements under the same conditions of current.

## 6. Discussion and Conclusions

The induction planar actuator has potential in terms of application to the inspection of inclined ferromagnetic surfaces, such as ship hulls, tanks, pipelines and other sorts of metallic surfaces. Other systems already use magnetic attractive force to keep the device attached to the hull or other ferrous plates. The large normal force produced is highly advantageous and it allows keeping the device attached to the surface. The planar motion produced from only one device reduces the need for several actuators for orthogonal motion which decreases the weight and size of the system. In terms of inspection of non-ferromagnetic plates, the application of the actuator can be made in horizontal surfaces, since an adhesion force is not produced. In case 3, although an attraction force is present, the thrust force is not as high as that produced in case 1. The calculations and studies performed show that the thrust force produced by the induction planar actuator may be too small to move the inspection platform along heavily inclined surfaces, which could limit the application to the inspection of inclined ferromagnetic surfaces in the ascendant direction, allowing its utilization only when the movement is descendant. Another problem may be overcoming welds or strong roughness in the surface. 

A comparison of the magnitude of forces produced with the different secondaries as analysed shows that the best case occurs with the double layered secondary. In case 3, the lower conductivity of the steel causes a reduction of the thrust force, while in case 2, the lack of a low reluctance magnetic circuit in the secondary reduces the magnetic flux entering the secondary, as seen in Figure 7b, and thus decreasing both the thrust and normal force magnitudes. The repulsive normal force produced in case 2 is even negligible when compared to the weight of the device. In case 3, the magnetic air-gap is 2-mm thick and a higher normal force is presented when compared to case 1, which has a 3-mm thick magnetic air-gap.

The numerical and analytical results of the distribution of the magnetic flux density calculated in the air-gap show good agreement for each case, as seen in Figure 6, Figure 7 and Figure 8. Each figure allows seeing the distortion in the distribution of the magnetic flux density in the air-gap due to the effect of the induced currents in the secondary. 

The results of the force obtained for case 1, Figure 9a,b, show good agreement. In the thrust force comparison, the average difference between the simulated and the measured values was 9.5% and between analytical and measured values, 5.2%. The normal force difference was 7.2% between the simulated and the measured values and −11.0% between analytical and measured values. Regarding case 2, the average difference between the numerical and the measured values was 10% and between analytical and measured values, 6.7%. The normal force was not measured, because the load cell does not have sensitivity suited to the values of levitation force produced when only the aluminium plate forms the secondary. Numerical and analytical values of the normal force for case 2 are presented and they do not show good agreement. With relation to case 3, the average difference between numerical and measured values of thrust force was 18.5% and between analytical and measured values, −26.2%. The normal force difference was −2.2% between the simulated and the measured values and −11.9% between analytical and measured values. This was due to experimental errors produced by thrust force acting on the normal force measurement (*i.e.*, on the load cell). This interferes with the load cell measurement and also changes the air-gap; the thrust force pushes the car, increasing the air-gap at one side and reducing it on the other side. Since the thrust force is critical when considering the movement on an inclined surface, the excellent fit between the measured and theoretical values allows a more accurate study to be carried out using the finite element model and design modifications. 

The theoretical models show good computation of the produced forces, and will help in further analysis. The analytical model allows understanding the behaviour of the magnetic flux density in the air-gap and in the conductive components of the secondary and calculating the forces. 

In Section 3.1 a set of equations was presented for calculating the fields when the actuator under study has two plates in the secondary, and, in Section 3.2, a set of equations that can be used when the secondary has only one sort of plate. The equations presented in Section 3.1 can also be applied to an actuator with only one material in the secondary, considering that the layer under the plate is air. However, the equations of Section 3.2 have a short form, when compared to the equations of Section 3.1. This characteristic is advantageous when the equations of parameters of the equivalent circuit are developed. In this work, the authors verified that when only a thin layer of aluminium was presented in the secondary, as in case 2, the analyses of the actuator by means of the equations of Section 3.1 produces results that are closer to the measured ones. It was also verified that the equations of Section 3.2 produced results with a considerable error in relation to the measured values in case 2. So, the results of the magnetic flux density and force shown in Figure 7, Figure 11 and Figure 12 where obtained by means of Equation (11) to Equation (19).

Comparing the theoretical models developed for this work, the numerical model has the advantage of predicting the conditions of the magnetic field in the ferromagnetic core of the primary. In the analytical model, the effects of the primary currents were taken into account by representing the windings as a linear current density (infinitely thin sheet). The equations of the analytical model lead to the fast computation of the fields and forces, while by the numerical model, the time of processing of each simulation is around three hours. The concomitant use of those two theoretical models and their results helps on the design of the actuator and on the analysis of the impact on the values of forces when modifications on dimensions were made and by applying different values of voltage and frequency to the windings of the primary.

The LIM end effects was not considered in the mathematical models presented in this paper because the authors analysed the behaviour of the actuator under static conditions (*s* = 1). According [12], the higher the velocity of the primary, the stronger the influence of the longitudinal end effects at the performance of the device. 

## Figures and Tables

**Table 1:** Dimensions of the induction planar actuator [11]. © 2015 IEEE. Reprinted, with permission, from IEEE Transactions on Magnetics.

Label	Description	Dimension
*t_coil_*	Coil thickness	3.75 mm
*l_coil_*	Coil length	18.5 mm
*l_yoke_*	Primary yoke thickness	14 mm
*l_tooth_*	Tooth length	45.3 mm
*l_trans_*	Tooth transition length	4 mm
*l_g-tooth_*	Tooth length (closer to the air-gap)	2.7 mm
*g_o_*	Air-gap length	2 mm
*d*	Aluminium layer thickness	1 mm
*t_fe_*	Ferromagnetic layer thickness	5 mm
*b_s-tooth_*	Tooth width	10 mm
*l_f_*	Tooth tip width (by the air-gap)	14 mm
*l_p_*	Tooth pitch	19.7 mm
*N*	Number turns per coil	180
